# Externalization of Mitochondrial PDCE2 on Irradiated Endothelium as a Target for Radiation-Guided Drug Delivery and Precision Thrombosis of Pathological Vasculature

**DOI:** 10.3390/ijms23168908

**Published:** 2022-08-10

**Authors:** Fahimeh Faqihi, Marcus A. Stoodley, Lucinda S. McRobb

**Affiliations:** Macquarie Medical School, Faculty of Medicine, Health and Human Sciences, Macquarie University, Sydney, NSW 2109, Australia

**Keywords:** arteriovenous malformation, drug targeting, endothelial cell, PDCE2, ionizing radiation, thrombosis, tumor targeting

## Abstract

Endothelial cells are highly sensitive to ionizing radiation, and exposure leads to multiple adaptive changes. Remarkably, part of this response is the translocation of normally intracellular proteins to the cell surface. It is unclear whether this ectopic expression has a protective or deleterious function, but, regardless, these surface-exposed proteins may provide unique discriminatory targets for radiation-guided drug delivery to vascular malformations or tumor vasculature. We investigated the ability of an antibody–thrombin conjugate targeting mitochondrial PDCE2 (E2 subunit of pyruvate dehydrogenase) to induce precision thrombosis on irradiated endothelial cells in a parallel-plate flow system. Click-chemistry was used to create antibody–thrombin conjugates targeting PDCE2 as the vascular targeting agent (VTA). VTAs were injected into the parallel-plate flow system with whole human blood circulating over irradiated cells. The efficacy and specificity of fibrin-thrombus formation was assessed relative to non-irradiated controls. The PDCE2-targeting VTA dose-dependently increased thrombus formation: minimal thrombosis was induced in response to 5 Gy radiation; doses of 15 and 25 Gy induced significant thrombosis with equivalent efficacy. Negligible VTA binding or thrombosis was demonstrated in the absence of radiation or with non-targeted thrombin. PDCE2 represents a unique discriminatory target for radiation-guided drug delivery and precision thrombosis in pathological vasculature.

## 1. Introduction

Vascular targeting with precision thrombosis is an approach aimed at occlusion and destruction of pathological vasculature in tumors and brain arteriovenous malformations (AVMs) [1,2,3]. This approach requires the identification of discriminatory biomarkers at the luminal surface of the pathological endothelium for use as molecular targets for the focused delivery of thrombogenic agents. To date, various endogenous markers have been identified on tumor vasculature and investigated in mouse tumor infarction models (reviewed in [1]). However, limitations to endogenous biomarker discovery in brain arteriovenous malformations (AVMs) have led to the use of ionizing radiation as a priming agent for target generation in this context [4,5,6,7,8,9,10,11,12,13].

Stereotactic radiosurgery can deliver a focused dose of radiation to clustered vessels in an AVM nidus, allowing precise target expression for radiation-guided drug delivery [14]. Radiation damage sets off a cascade of cellular events in endothelial cells that can alter the molecules or proteins at the cell surface (surfaceome) [4,5,15,16]. Surface exposure of common inflammatory markers such as phosphatidylserine (PS), vascular cell adhesion molecule 1 (VCAM-1) and intercellular adhesion molecule 1 (ICAM-1) have been previously investigated as radiation-stimulated targets in an AVM animal model [6,7,13,17]. However, their frequent exposure in response to other biological stimuli may reduce targeting specificity. Interestingly, canonical autophagic pathways that normally process damaged molecules via the lysosome appear to become overwhelmed after radiation exposure, leading to the non-canonical transport of intracellular proteins to the extracellular space or cell surface [18]. Functionally, this may represent a survival mechanism for the removal of toxic proteins or a signal (exposure of “self” antigens) to alert the immune system to the presence of stressed or damaged cells [19]. Regardless, these normally intracellular proteins may represent highly discriminatory markers for targeting on the surface of radiation-primed vasculature, given their absence on the surface of healthy vasculature and in vessels exposed to other biological stimuli. One such protein is the E2 subunit of pyruvate dehydrogenase (PDCE2). This mitochondrial protein can translocate from intracellular compartments to the endothelial surface in response to high doses of radiation via this unconventional pathway [4,18]. The lack of surface exposure of PDCE2 in healthy tissues may make this an ideal target. To date, ectopic PDCE2 exposure has only been described in primary biliary cirrhosis, an autoimmune disorder causing the inflammation and destruction of the intrahepatic ducts [20,21,22].

The primary objective of this study was to assess PDCE2 as a molecular target for radiation-guided delivery of pro-thrombotic agents. A vascular targeting agent (VTA) was designed to deliver the pro-coagulant thrombin to PDCE2 exposed at the endothelial surface after irradiation. An anti-PDCE2 antibody was chemically linked to thrombin to create a “coaguligand” as VTA [3]. VTAs were tested in vitro for focused thrombosis induction using a parallel-plate flow system where whole human blood is circulated in a pulsatile manner over irradiated endothelial monolayers. This system is an economical and ethical way to assess VTA specificity and efficacy before translation to animal models and has been used successfully to assess other radiation-induced markers and associated pro-thrombotic VTAs [11,12]. Using this system, we demonstrate that the endothelial surface expression of PDCE2 after 15–25 Gy of radiation is sufficient to bind targeting antibodies under a high flow and confirm that the antibody-directed delivery of thrombotic effectors to ectopic PDCE2 can induce selective thrombosis on irradiated human endothelial cells.

## 2. Results

### 2.1. Anti-PDCE2 Antibody Binds Specifically to the Irradiated Cell Surface under Flow

**Figure 1:**
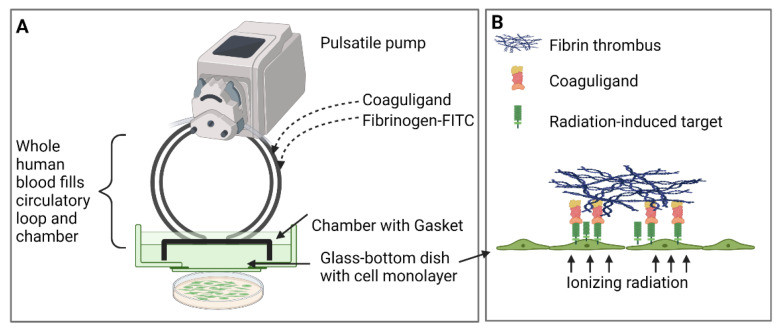
Schematic figure of the parallel-plate flow system. (**A**) A closed-loop pulsatile parallel-plate flow system is assembled using a chamber with a gasket that allows whole human blood or growth medium to flow dynamically over an endothelial monolayer grown on a glass-bottomed Petri dish. Cells are irradiated at set time points before construction of the system. Coaguligand or fluorescent conjugates are added to the circulation as the vascular targeting agents (VTAs). (**B**) Fibrin thrombus formation occurs over the irradiated cells after administration of fluorescently labelled fibrinogen and coaguligand to the circulation. Figure created using biorender.com.

A parallel-plate flow system was established, as previously described, to allow circulation of whole human blood or growth medium over a confluent layer of human endothelial cells after irradiation or sham treatment (Figure 1) [11,12].

**Figure 2:**
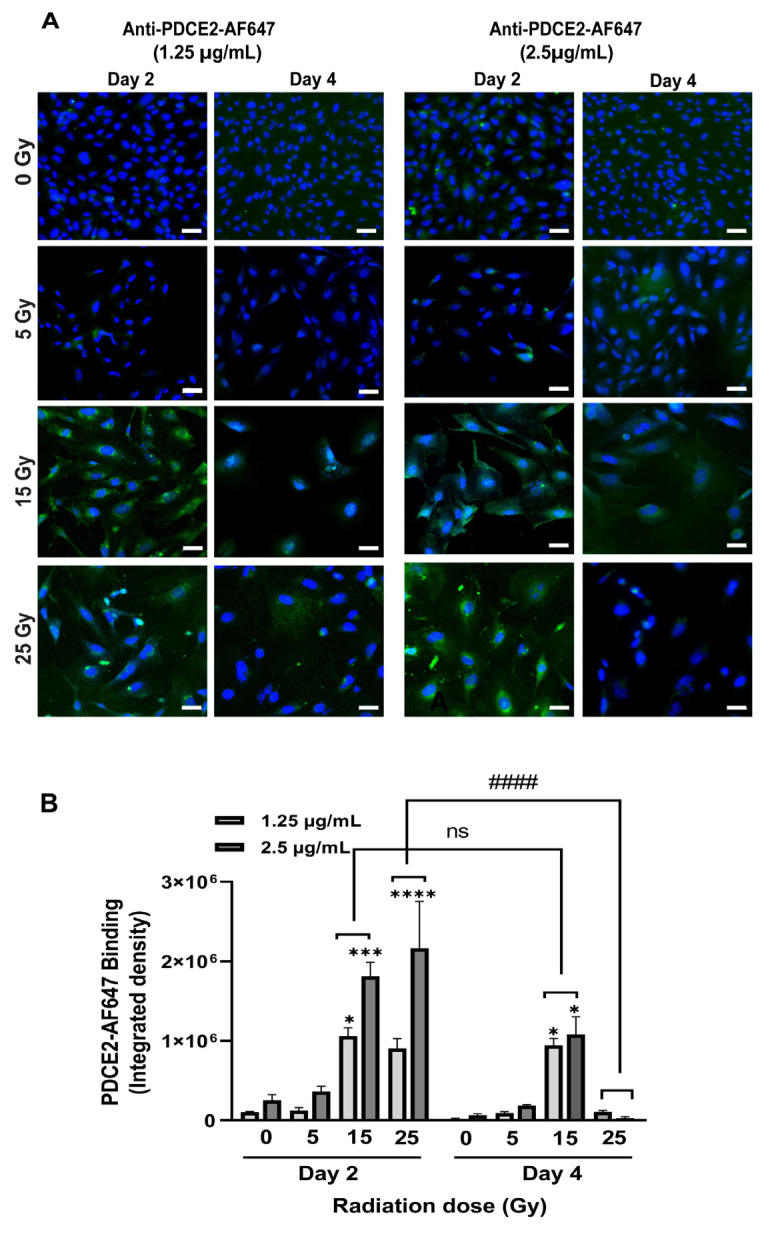
Anti-PDCE2 antibodies bind to the surface of irradiated endothelial cells under optimized shear flow in a parallel-plate flow system. (**A**) Confocal images demonstrate binding of fluorescently tagged PDCE2 antibody (AF-647, green) at two different concentrations (1.25–2.5 µg/mL) to the irradiated cell surface at day 2 and day 4 post-irradiation (0–25 Gy) after 15 min of circulation in growth medium in the parallel-plate flow system. Nuclei were stained with DAPI (blue). Magnification 200× (Bar = 50 µm). (**B**) Confocal images from three independent experiments were analyzed using NIH Image J. Data (integrated density) are shown as mean ± SEM and were analyzed using two-way ANOVA followed by Tukey’s post-hoc analysis. * *p* < 0.05, *** *p* < 0.001, **** *p* < 0.0001, compared to corresponding non-irradiated cells. #### *p* < 0.0001, comparison between timepoints. ns, not significant.

Vascular targeting agents (VTAs) comprising ligands (antibody) recognizing PDCE2 without a pro-thrombotic component were chemically conjugated to fluorophores (Alexa Fluor 647) and injected into the flow system in the presence of growth medium. Two concentrations of tagged antibodies (1.25 and 2.5 µg/mL) were added to the circulation, and binding to the cell surface was assessed on endothelial cells exposed to radiation doses (0, 5, 15, 25 Gy) either 2 or 4 days prior. After 15 min of flow circulation, cells were fixed, and fluorescence intensity measured to evaluate antibody binding and target expression. It was observed that both radiation dose and time of administration post-treatment affected the PDCE2 surface binding under flow (Figure 2). The most significant level of attachment was observed following a dose of 25 Gy radiation at day two, but this was entirely diminished by day four. Cells that received a dose of 15 Gy also demonstrated significant PDCE2 surface exposure at day two; however, this expression persisted through day four. No significant increase in signal was noted with the lowest radiation dose (5 Gy) at either timepoint. Non-irradiated cells demonstrated little binding. Increasing the concentration of VTA did not induce any significant change in surface-bound signal in these experiments.

These data show that PDCE2 can be targeted at the cell surface with precision in response to radiation doses of 15 and 25 Gy. The transient peak observed at day two with the higher radiation dose may reflect a rapid peak in non-canonical autophagic processing that is quickly resolved [18]. Lower radiation doses may stimulate a reduced but prolonged autophagic response, resulting in sustained PDCE2 exposure at the surface.

### 2.2. Targeted PDCE2 Coaguligands Increase Fibrin Clot Formation on Irradiated Cells under Flow

Having demonstrated with fluorescently tagged antibodies that PDCE2 can be targeted at the cell surface under high flow, a click chemistry approach was utilized to conjugate purified anti-PDCE2 antibody to human thrombin. This coaguligand was then used as the VTA to assess selective thrombogenic activity in the presence of circulating human whole blood. Cell monolayers were incorporated into the flow system and subjected to whole blood circulation on day 2 after irradiation (0, 5, 15, 25 Gy). Thrombus formation was evaluated by incorporating FITC-tagged fibrinogen into the circulating blood and microscopically measuring fibrin clot deposition in the presence of the VTA at final concentrations of 2.5 and 5 µg/mL or free thrombin at 2.5 µg/mL (equivalent activity to a ~10 µg/mL VTA dose). The total thrombus volume per field of view was assessed to quantitate the efficacy of clot formation. The average volume of individual clots per field of view was determined to monitor potential changes in fibrin cross-linking and clustering as a measure of thrombus stability.

**Figure 3:**
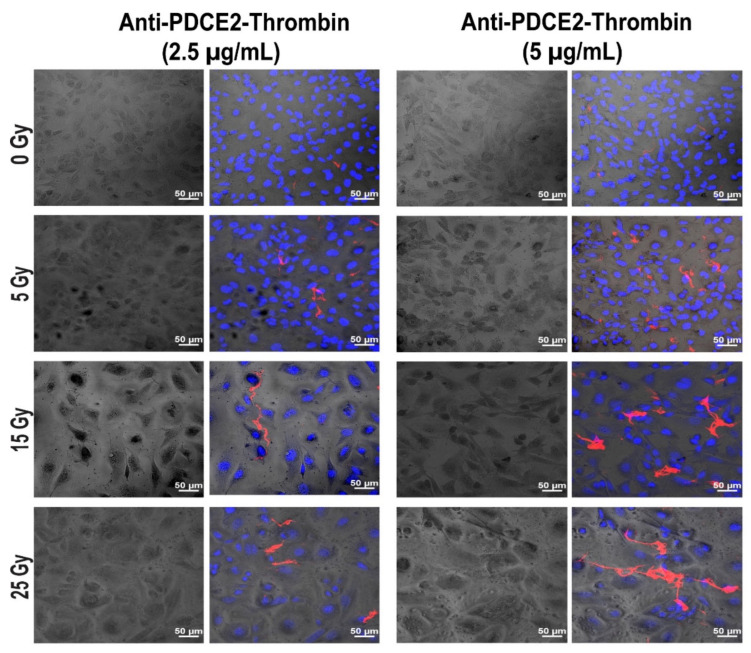
PDCE2-targeting coaguligand induces selective fibrin clot formation on irradiated cells in a parallel-plate flow system. Representative confocal images showing FITC-tagged fibrinogen (red) forming fibrin clots on confluent endothelial monolayers at day 2 post-irradiation (0, 5, 15 and 25 Gy) in the parallel-plate flow system containing whole human blood. Anti-PDCE2 antibody–thrombin coaguligands were injected into the circulation at concentrations of 2.5 and 5 µg/mL for 15 min. Nuclei were stained with Hoechst (blue). Cells were imaged with phase contrast microscopy. All images were captured at a magnification of 200× (Bar = 50 µm). It should be noted that in response to radiation, cell layers retain full confluence; however, as they undergo significant hypertrophy (cell and nuclear enlargement), fewer cells are observed per field of view.

**Figure 4:**
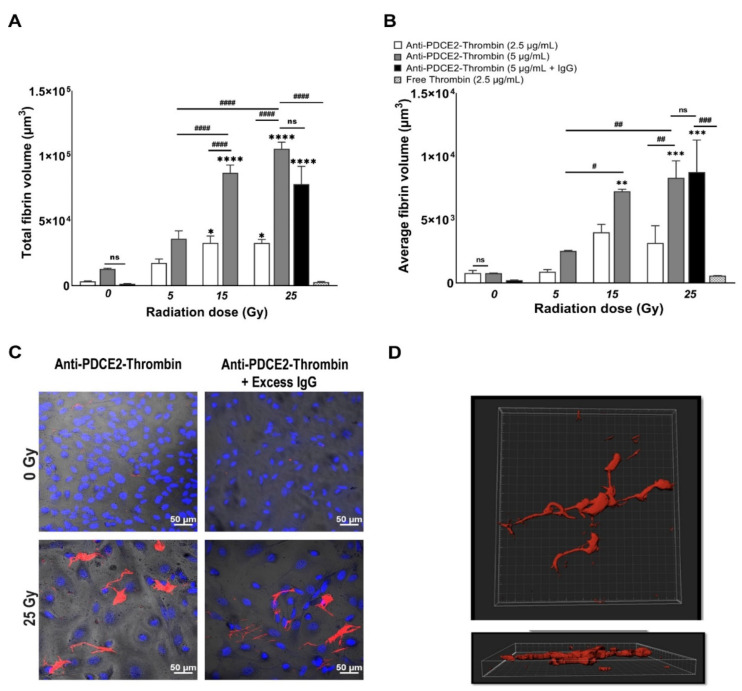
Fibrin clot formation is dependent on radiation dose and coaguligand concentration. Total (**A**) and average (**B**) fibrin volume per field of view were quantified by IMARIS after 3D volume rendering of fibrin clots. Total fibrin volume represents all fluorescence captured per field of view; average fibrin volume represents the sum of the fluorescence of each individual fibrin clot divided by the total number of clots per field of view. All thrombosis assays in the parallel-plate flow system were performed at day 2 after radiation exposure (0, 5, 15, 25 Gy) in the presence of coaguligand (2.5 or 5 µg/mL), or thrombin (2.5 µg/mL, equivalent activity), with or without excess non-specific IgG (12.5 µg/mL). Data are shown as mean ± SEM (at least 3 independent experiments) and were analyzed by two-way ANOVA with Tukey’s post-hoc analysis. * *p* < 0.05, ** *p* < 0.01, *** *p* < 0.001, **** *p* < 0.0001, comparison to corresponding non-irradiated cells. # *p* < 0.5, ## *p* < 0.01, ### *p* < 0.001, #### *p* < 0.0001, comparison within and between radiation groups. ns, not significant. (**C**) Representative confocal images showing fibrin clot formation of FITC-tagged fibrinogen (red) with and without excess non-specific IgG antibody in the presence of anti-PDCE2-thrombin coaguligand (2.5 µg/mL). Experiments were performed only with the highest coaguligand and radiation dose at day 2. Nuclei were stained with Hoechst (blue). Magnification = 200× (Bar = 50 µm). (**D**) Representative images of fibrin clots (red) visualized on endothelial layers using confocal microscopy after 3D reconstruction of Z-stacks (volume rendering) using IMARIS.

It was observed that fibrin clot formation was dependent on both radiation and VTA dose (Figure 3 and Figure 4). In general, the total and average volume of fibrin clots demonstrated similar trends. Compared with non-irradiated control cells, a dose of 25 Gy radiation induced a significant increase in total fibrin volume at both VTA concentrations of 2.5 and 5 µg/mL (~10- and 8.5-fold, respectively) and enhanced average fibrin volume (~10-fold) at 5 µg/mL of VTA. Interconnected fibrin clot structures were also visualized on cell layers exposed to 25 Gy radiation with 5 µg/mL VTA. At 15 Gy, a significant enhancement in total fibrin volume was noted using both 2.5 and 5 µg/mL VTA compared to non-irradiated control (~10- and 7.5-fold, respectively); however, the average volume increased significantly (~10-fold) only at the higher VTA concentration. Increasing the radiation dose from 15 to 25 Gy did not significantly increase fibrin clot formation. At 5 Gy, neither concentration of VTA significantly increased the total and average fibrin volume compared to corresponding controls. In this group, fibrin was only deposited in a diffuse pattern. Negligible clot formation occurred in the absence of radiation. Non-targeted, free thrombin at an equivalent activity level demonstrated negligible total and average fibrin volume on irradiated cells and was comparable to the non-irradiated control group. These data similarly demonstrate the significant binding of a PDCE2-targeting VTA within the 15 to 25 Gy dose range. The targeted delivery of thrombin can induce localized thrombosis in a precise manner.

### 2.3. Excess Non-Specific IgG Does Not Inhibit Clot Formation

Fc receptors can be expressed in response to post-radiation inflammatory processes and could contribute to non-specific binding to the irradiated surface when VTA constructs incorporate whole antibodies [23]. Thus, a further set of experiments were performed in the parallel-plate flow system that included injection of a 5- to 10-fold molar excess of non-specific IgG (12.5 µg/mL) to block potential non-specific interactions with Fc receptors (Figure 4). These experiments were only performed with the highest concentration VTA (5 µg/mL) and radiation dose (25 Gy). It was observed that total and average fibrin volumes remained statistically unchanged at both 0 and 25 Gy in the presence of excess IgG compared to corresponding experiments performed without IgG. Both total and average fibrin volumes were still enhanced significantly (57- and 40-fold) at 25 Gy with respect to non-irradiated controls following IgG binding. A reduction to zero thrombosis was detected in non-irradiated cells following IgG circulation. No visible changes were observed in clot complexity between the IgG-treated and untreated groups. These results demonstrate that non-specific binding to Fc receptors is minimal in this system and that the thrombosis observed is a result of PDCE2-specific targeting.

### 2.4. High Throughput Static Systems Are Suitable for Preliminary VTA Assessment

This, and earlier studies [11,12], demonstrate the utility of using the parallel-plate flow system as an ethical approach to VTA testing that allows the assessment of conjugate efficacy before pre-clinical animal studies, as well as pre-testing in the human context (human cells, human blood). However, the methodology remains limited by the volume of human blood and human participants required to run each experiment. Thus, a second aim of this study was to determine whether a high-throughput approach could be developed with a similar sensitivity to the parallel-plate flow system for the pre-screening of VTAs. Two systems were thus developed and assessed: a static Petri dish model and a static multi-well model.

#### 2.4.1. Static Petri Dish Model

**Figure 5:**
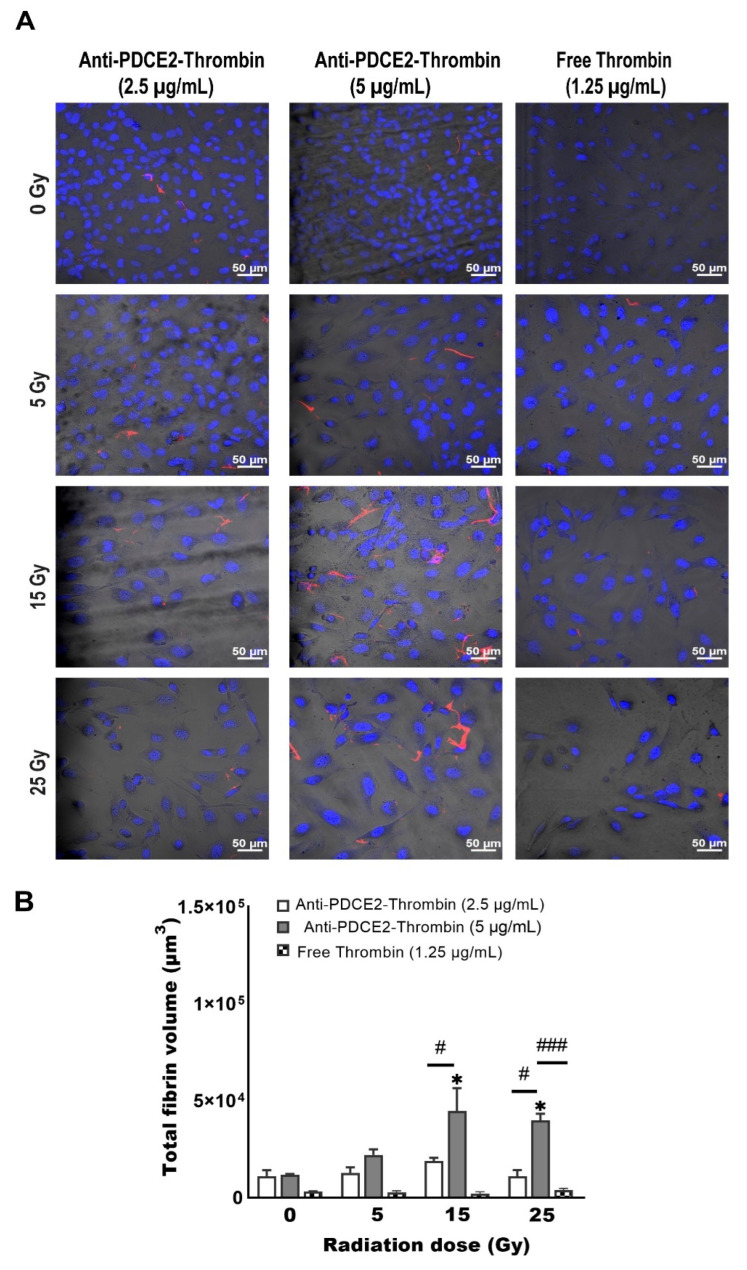
Fibrin clot formation is dependent on coaguligand concentration and radiation dose under static conditions (static Petri dish model). (**A**) Representative confocal images of FITC-tagged fibrinogen (red) forming fibrin clots on irradiated cells (0, 5, 15, 25 Gy) at day 2. Anti-PDCE2 antibody–thrombin coaguligands (2.5 or 5 µg/mL) or free thrombin (1.25 µg/mL, equivalent activity) were added to confluent cell layers in 2 mL of whole human blood for 10 min before washing, confocal imaging and 3D rendering. Nuclei were stained with Hoechst (blue); all images were taken at magnification 200× (Bar = 50 µM). (**B**) Total fibrin volume per field of view was quantified by IMARIS. Data are shown as mean ± SEM (3 independent experiments) and were analyzed by two-way ANOVA with Tukey’s post-hoc analysis. * *p* < 0.05, comparison to corresponding non-irradiated cells. # *p* < 0.05, ### *p* < 0.001, comparison within radiation groups.

In this model, human endothelial cells were cultured as per the parallel-plate flow study in 35 mm glass-bottom pre-coated Petri dishes and irradiated at 0, 5, 15 or 25 Gy. However, only 2 mL of fresh human blood containing FITC-fibrinogen, VTA or free thrombin was prepared and pipetted directly onto the cell layer. After 5, 10, or 15 min, cells were washed, fixed, stained and imaged for 3D reconstruction. After 15 min of blood incubation, excessive clotting was observed regardless of radiation dose, while no visible clot was observed after 5 min (data not shown). It was established that 10 min was optimal for the induction of specific thrombus formation in this static system. At this timepoint, a significant enhancement in total fibrin clot was observed at both 15 and 25 Gy with 5 µg/mL VTA in comparison with 0 Gy controls (~4- and 3.5-fold) (Figure 5). No significant thrombosis was observed at the lower radiation dose (5 Gy) with either 2.5 µg/mL or 5 µg/mL VTA, or in the absence of radiation. Free thrombin at an equivalent activity level did not induce any significant thrombus development at any radiation dose. Overall, total fibrin volume demonstrated a similar trend to that observed in the parallel-plate flow system; however, the maximum volume measured was approximately 50% lower.

#### 2.4.2. Static Multi-Well Model

To further reduce the volume of blood and VTA required in pre-testing studies, a 96 multi-well plate model was used. As performed in the parallel-plate flow system, FITC-fibrinogen was added to recalcified blood with VTA at concentrations of 0, 2.5, 5 or 10 µg/mL. A volume of 200 µL of blood was added directly to each well and incubated for 10 min before washing and imaging. Fluorescent images of each well were captured directly with an EVOS Cell Imaging System, while the FITC signal in each well was quantitatively determined in a microplate reader. These studies were only performed at a dose of 25 Gy.

**Figure 6:**
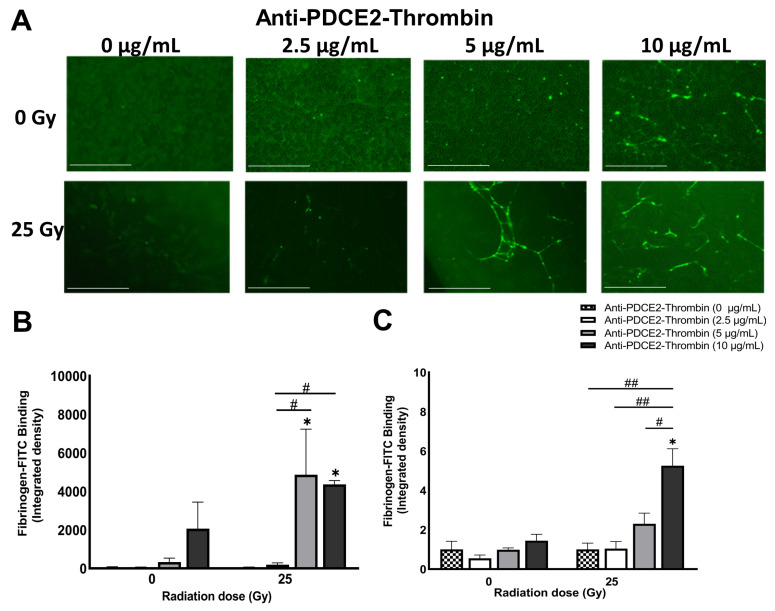
High throughout, static approaches for assessment of fibrin deposition demonstrate lower sensitivity (static multi-well model). (**A**) Representative fluorescent images of a 96-well microplate demonstrating extent of fibrin clot deposition (FITC, green) 2 days after irradiation (25 Gy only) in the presence of anti-PDCE2-thrombin coaguligand (0–10 µg/mL) and 200 µL whole human blood for 10 min. Images were captured using an EVOS Imaging System. Magnification, 100× (Bar = 400 µm). (**B**) FITC fluorescence (integrated density) in fluorescent images was analyzed using NIH Image J. (**C**) FITC-labelled fibrinogen binding was assessed in the same plates using a fluorescent microplate reader. For both analyses, average value of triplicate wells per individual treatment group is shown ± SEM (one independent experiment). Data were analyzed using two-way ANOVA with Tukey’s post-hoc analysis. * *p* < 0.5, comparison relative to non-irradiated, saline-treated cells (no coaguligand). # *p* < 0.05, ## *p* < 0.01, comparison within radiation group.

No visible fibrin clot was detected in the absence of VTA (Figure 6). Negligible thrombosis was observed in both irradiated and non-irradiated cells treated with 2.5 µg/mL of VTA. Significant thrombosis was observed on irradiated cells at the higher VTA concentrations (5 and 10 µg/mL) only. At 10 µg/mL, non-specific activation of the coagulation cascade and clot formation was visibly induced on non-irradiated cells; however, this increase was not statistically significant. The measurement of FITC fluorescence showed a higher background signal when the raw data was considered for non-irradiated cells compared to irradiated cells; however, when normalized to the respective saline-treated controls, clear trends were seen within each radiation dose group. Overall, while this model demonstrated similar binding trends; the sensitivity of this static model (concentration of coaguligand required) was significantly lower than that of the dynamic flow system and the static Petri dish model.

## 3. Discussion

In this study, a parallel-plate flow system was employed to systematically examine cell surface PDCE2 exposure and test an anti-PDCE2-thrombin coaguligand as a VTA for selective and stable thrombosis on irradiated cells. The PDCE2-targeting VTA provided acceptable activity in stimulating clot formation on irradiated human endothelial cells with whole blood circulating under flow conditions. Using this strategy, the following major findings have been concluded: (1) PDCE2 antibody can be effectively bound to the surface of irradiated cells and maintained under flow conditions, confirming cell surface ectopic expression of this target on human cells in response to high radiation doses (15–25 Gy); (2) the developed anti-PDCE2-thrombin coaguligand can specifically and selectively target externalized PDCE2 and maintain the enzymatic activity of thrombin; and (3) the combination of ectopic PDCE2 expression level and targeted thrombin activity is sufficient to induce significant thrombosis on irradiated cells with minimal off-target effects on non-irradiated endothelial cells and minimal thrombosis in the presence of free, non-targeted thrombin. These data support the hypothesis that ectopic PDCE2 exposure induced in response to ionizing radiation provides a putative discriminatory target for radiation-guided drug delivery, and that the accumulation of PDCE2-targeted thrombogens can overcome endothelial defenses to induce focused thrombosis on pathological vasculature.

The parallel-plate flow system was previously established for the in vitro testing of externalized phosphatidylserine (PS) and alpha-B-crystallin (CRYAB) as radiation-induced surface markers [11,12]. This dynamic system represents an ethical and economical approach for the rapid assessment of multiple VTA constructs prior to pre-clinical animal studies. It allows the assessment of VTA binding and thrombosis induction under pulsatile flow, conditions more closely replicating physiological parameters important for thrombus formation that cannot be replicated in static models. This system also allows the pre-assessment of targets and VTAs in a model more relevant to the human context (human cells, human blood). In line with earlier studies, we found that maximum thrombosis in response to PDCE2-targeting VTAs occurred at the higher 15 and 25 Gy doses [11,12]. Clinical median marginal dosage required to achieve AVM closure using stereotactic surgery with minimal adverse radiation effects is typically 20 Gy (19–22 Gy), close to the upper 25 Gy limit used here [24,25,26], so target expression is achievable within clinically relevant dose ranges. The potential ability to stimulate the expression of these targets and thrombosis at lower marginal radiation doses (e.g., 15 Gy), as observed here, is an important result. SRS suffers from significantly reduced obliteration rates at marginal doses of 15–16 Gy (60–70%), while higher doses of radiation increase the risk of late adverse effects and clinical complications, limiting the application of radiosurgery, particularly for complete obliteration of large AVMs [25,27]. In the clinic, this targeting approach could potentially allow lowering of the therapeutic radiation dose without a significant reduction in obliteration rates. Use of a lower dose may also increase the therapeutic window for PDCE2 targeting, as PDCE2 expression had greater durability (sustained expression) at the 15 Gy dose in this study relative to the 25 Gy dose. Further investigation of this phenomenon is warranted.

In contrast to the thrombosis observed at 15 and 25 Gy doses of radiation, thrombosis was not significantly induced at the lowest radiation dose employed when targeting either PDCE2, in this study, or CRYAB, previously [12]. Limited binding at 5 Gy could reduce the chance of off-target thrombus formation following coaguligand presentation both in the systemic, non-irradiated vasculature, as well as in adjacent vessels that may receive a minimal dose. Interestingly, a PS-targeting VTA was observed to be significantly more effective at this 5 Gy dose in the same parallel-plate flow system [11]. PS is an abundant phospholipid that flips from the inner surface of the plasma membrane to the outer surface in response to a variety of biological stimuli, including radiation, and is most often observed in apoptotic cells [28,29,30]. The higher efficacy may reflect greater levels of PS exposure at the surface relative to PDCE2 and CRYAB or, alternatively, could reflect the relative affinity of each ligand for their respective targets [1]. A recent in vivo investigation of PS targeting in a rat AVM model revealed that both irradiated and non-irradiated vasculature could be thrombosed with a high affinity PS-targeting VTA [17]. Off-target thrombosis in this instance could be minimized with reduced VTA dosing; however, the possibility of unintended thrombotic occlusion caused by non-specific PS expression in off-target vasculature must be addressed in the future optimization of this target. Future studies could examine lower affinity PS ligands to assess whether specificity could be improved. For the targeting of intracellular proteins such as PDCE2 and CRYAB, lower basal levels of expression after radiation relative to PS may lower the risk of off-target binding but may also reduce in vivo efficacy. Overall, the current study supports advancement of this target and associated VTA to pre-clinical animal studies to assess in vivo efficacy and specificity. This target could be examined in animal models of brain AVMs as previously described [17] and in mouse models of cancer where radiation was first described to enhance tumor vascular targeting [31].

One limitation of the parallel-plate flow system used here is the volume of blood used for each independent experiment, which necessitates the constant recruitment of donors. This slows progress when multiple targets and VTAs are to be analyzed. Hence, a second goal of this study was to establish whether a static in vitro model of thrombosis induction could be developed as an efficient method of VTA assessment before the hemodynamic flow studies. A static Petri dish assay allowed a reduction in the amount of blood used to one-sixth of the original volume (2 mL vs. 12 mL). Although this model was not as sensitive as the flow system to differentiate radiation dose and coaguligand concentrations, it demonstrated similar trends: the highest level of thrombosis was detected at 15 and 25 Gy with a higher coaguligand dose; no fibrin clot formation was found with an equivalent activity dose of free thrombin or on non-irradiated cells. When compared to the parallel-plate flow findings, the amount of clot identified using the static model was considerably lower; however, this was not unexpected given that cells had less access to the clotting factors, fibrinogen and VTA and given the lower blood volume used. Reduced incubation duration (10 vs. 15 min) may also have influenced fibrin deposition. Lower levels of thrombosis may also indicate how shear stress in the flow system contributes to the formation and maturation of the clot (e.g., from platelet activation), which was not fulfilled via the static model [32]. Overall, this static assay offers some advantages over the flow model, such as reduced blood volume, coaguligand consumption and experimental time. While the need for confocal imaging is still laborious and time-consuming, this static Petri dish model could be used to increase the productivity of preliminary screening where multiple radiation-induced targets and VTAs are assessed simultaneously.

The static multi-well model proved not as sensitive and reproducible as the Petri dish system to differentiate radiation dose and coaguligand concentrations and cannot be used as a single technique to monitor fibrinogen deposition with the current set-up. Several technical issues were encountered with this model. The handling of viscous blood samples in small volumes led to pipetting inaccuracy, while in the presence of high clot formation, removing loosely bound blood after incubation was problematic as increased washing caused greater a detachment of cells (and clots) from the surface, negatively affecting accuracy. Further optimization of this approach is necessary before use as a high-throughput assay; however, the promising trend observed with the microplate fluorescent reading suggests feasibility with further methodological development.

In conclusion, the lack of surface localization of mitochondrial PDCE2 on normal healthy endothelial cells, and the increased surface expression established here in response to radiation, supports further investigation of PDCE2 as a discriminatory surface marker for vascular targeting in radiation primed tissues. Overall, these data indicate that using a lower radiation dose (15 Gy versus 25 Gy) in the clinic could provide a larger therapeutic window for VTA administration with PDCE2 as a target and allow for more specific targeting without off-target binding and thrombosis. The static and parallel-plate flow systems described here provide economical and ethical models for rapid comparisons of multiple radiation-stimulated targets and VTA constructs prior to pre-clinical evaluation in animal models. The data gained from these model systems support the advancement of this target and associated VTA to pre-clinical studies to examine precision thrombosis in both irradiated tumor and AVM animal models.

## 4. Materials and Methods

### 4.1. Cell Culture and Irradiation

The immortalized human cerebral microvascular endothelial cell line, hCMEC/D3 (CELLutions Biosystems Inc., Burlington, ON, Canada), was cultured in a complete medium consisting of Endothelial Basal Medium-2 (EBM-2) (Lonza, Basel, Switzerland), fetal bovine serum (5%), penicillin-streptomycin (1%), HEPES (10 mM) (Thermo Fisher, Waltham, MA, USA) and human basic fibroblast growth factor (1 ng/mL) (Sigma-Aldrich, St. Louis, MI, USA), maintained at 37 °C carbon dioxide 5% in humidified 95% air [18]. All cells between passages 11 and 25 were sub-cultured by trypsin-EDTA solution (Sigma-Aldrich). Trypan blue staining via an automated cell counter (Thermo Fisher) determined the viability and number of cells. All dishes were pre-coated with 100 μg/mL rat tail collagen IV (In Vitro Technologies, Sydney, NSW, Australia). One mL of collagen diluted in autoclaved water was added to each Petri dish, followed by at least 1 h incubation and was then washed three times with phosphate-buffered saline (PBS). Cells were seeded at 1 × 10^5^ cells/mL in pre-coated 35 mm glass-bottom Petri dishes (MatTek Corporation, Ashland, MA, USA) containing 2 mL complete EBM-2 medium, which was replaced with fresh medium every other day. Confluent cells (100%) were irradiated (0–25 Gy) by a linear accelerator (LINAC) (Crawley, UK) at Macquarie University Hospital (Sydney, Australia) as previously described [11,12]. Sham- irradiated cells were treated identically but without radiation.

### 4.2. Fluorescent Antibody Conjugate Preparation

Labelled anti-PDCE2 antibody was prepared by conjugating Alexa Fluor 647 (AF-647) (Thermo Fisher) to a PDCE2 mouse monoclonal antibody (sc-166899, Santa Cruz, CA, USA). Briefly, 100 µg of antibody was purified using NAb Protein G Spin Columns (Thermofisher) to eliminate gelatin. Purified anti-PDCE2 antibody (1 mg/mL) was resuspended in sodium bicarbonate (pH 8.0) before mixing with dye in dimethyl sulfoxide. The mixture was kept for 1 h at room temperature with gentle agitation. The absorbance of antibodies and dye was measured via NanoDrop (Thermo Fisher). The degree of labelling (DOL) was determined as 1.2.

### 4.3. Antibody–Thrombin Coaguligand Preparation

Click-&-Go™ Lys-to-Lys Protein-Protein Conjugation Kit (Click Chemistry Tools, Scottsdale, USA) was used to conjugate purified anti-PDCE2 antibody (166899, Santa Cruz, CA, USA) and human thrombin (Jomar Life Research, Scoresby, VIC, Australia). Based on the manufacturer’s protocol, 121 µL thrombin (3.57 mg/mL) and 100 μL anti- PDCE2 antibody were labelled with a 20-fold molar excess of TCO and Tetrazine reagents, respectively. The labelled anti-PDCE2 and thrombin were mixed and kept at room temperature for 1 h. The efficiency of the conjugation was evaluated by SDS-PAGE. The ratio of conjugation measured via NIH Image J (Bethesda, Maryland, USA) [33] was 60%.

### 4.4. Thrombin Activity Assay

The enzymatic activity of thrombin was compared before and after the conjugation process using a fluorometric thrombin activity assay (SensoLyte 520, Ana Spec, Fremont, CA, USA). To examine the activity level, 10 μL of free thrombin and conjugated thrombin were diluted in 40 μL buffer and assayed for excitation/absorbance at 485/535 nm. The total thrombogenic activity of the coaguligand was determined to be approximately 61% of the original thrombin (equivalent to 1940 NIH units/mg).

### 4.5. Human Blood Collection

Human blood was collected with informed consent from healthy volunteers. The research was authorized by the Macquarie University Human Research Ethics Committee (approval number HREC: 5201837496785). Healthy adult volunteers were recruited in this study with an age range of 25–58 years. Written consent was obtained from all volunteers. A volume of 20 mL was taken intravenously based on phlebotomy guidelines. The blood was collected into 4.5 mL anti-coagulation CTAD tubes (BD Bioscience, Sydney, NSW, Australia). The initial 3–5 mL of blood was discarded to avoid contamination with tissue factor or thrombin [34]. The collected blood was kept at room temperature up to 30 min before applying it to the flow system.

### 4.6. Parallel-Plate Flow System Set-Up and Operation

The parallel-plate flow system was set up as originally described [11,12]. All flow system parts were assembled after rinsing with distilled warm water based on Series 1400 Pulsatile Blood Pumps User’s Manual (Harvard Apparatus, Cambridge, MA, USA). The average shear stress of 3.1 dynes/cm^2^ and constant flow rate were achieved by setting a rate adjustment of 38 strokes/min, stroke volume of 2.4 mL/min and circulation time of 15 min. These flow conditions were previously established to simulate in vivo experiments while preserving the confluence of cells during blood circulation with minimum cell lifting [11]. Approximately 11–12 mL of whole human blood was recalcified by adding 5 mM calcium chloride and 10 mM magnesium chloride in a final volume of 1:10 dilution to the blood to restore the coagulation cascade. FITC-labelled fibrinogen (Molecular Innovations, Novi, MI, USA) with a final concentration of 130 µg/mL was incorporated into the whole blood immediately before circulation to visualize and measure fibrin clot formation. The mixture of recalcified, labelled blood (~12 mL) was injected into the enclosed circulation slowly but constantly to prevent blood bubble formation. In the next step, a collagen-coated 35-mm glass Petri dish with confluent cells following different doses of radiation were fitted in the gasket and connected to the pulsatile circulation tubes and vacuum inlet. The system was started with an optimized forwarding mode to avoid backward negative pressure. The anti-PDCE2-thrombin coaguligand (final concentration of 2.5 and 5 µg/mL) or non-specific IgG (final concentration of 12.5 µg/mL) was injected into the circulation immediately after starting the blood circulation. After 15 min of flow circulation, cells were disconnected from the pulsatile system and gently washed twice with PBS, followed by ethanol fixation (50% in PBS, 20 min), Hoechst staining (5 µg/mL, 5 min) and mounting for confocal imaging. Additionally, independent experiments were performed using non-conjugated free thrombin at a final concentration of 2.5 µg/mL (equivalent activity to 10 µg/mL of coaguligand based on percent reduction (39%) in thrombin activity post-conjugation and the size differential between the VTA (~180 kDa) and free thrombin (~30 kDa)).

### 4.7. Confocal Microscopy

Z-stack images were taken using a Zeiss LSM880 Confocal Microscope (Jena, Germany) with First/Last operational mode. The range and number of Z-stacks were adjusted based on FITC staining (8 slides). Images were acquired from three channels: bright field, ex358 nm/em461 nm for Hoechst (nuclei), and ex 495/em519 nm for FITC (fibrin clot) imaging. Two-dimensional confocal images were taken using two channels (ex358 nm/em461 nm, ex650 nm/em665 nm) for visualizing DAPI and AF-647 (PDCE2).

### 4.8. 3D and 2D Image Analysis

Thrombus volume was measured via 3D reconstruction of the Z-stack image using Bitplane (IMARIS software version 9) as previously described [11,12]. Briefly, a new surface was added to the Z-stack image via “add a surface” toolbar icon. The image contrast and brightness were adjusted, and the image was displayed in Blend mode. FITC was selected as the desired channel with absolute intensity set for thresholding. A threshold was manually adjusted to render all the fibrin structures. Finally, the volume of rendered thrombus was measured under the Statistics Tab. 2D images were analyzed with Zen 2.5 (Blue edition) software using identical settings between controls and irradiated cells (Carl Zeiss Microscopy, Germany). For analysis of AF-647 tagged antibody binding, the integrated density was determined per field of view using NIH Image J [33] after adjusting the threshold.

### 4.9. Static Models

#### 4.9.1. Static Petri-Dish Model

Human endothelial cells (hCMEC/D3) were cultured in 35 mm glass-bottom pre-coated Petri dish and irradiated at 0, 5, 15 or 25 Gy, as per the flow study method. At day 2 post-irradiation, 2 mL of fresh human blood, FITC-labelled fibrinogen and anti-PDCE2-thrombin conjugate (2.5 and 5 µg/mL) or free thrombin (1.25 µg/mL; an equivalent enzyme activity to 5 µg/mL conjugate) was added directly to the cells for 5, 10 or 15 min at room temperature. Cells were then thoroughly washed, fixed and stained before confocal imaging, 3D reconstruction and IMARIS analysis.

#### 4.9.2. Static Multi-Well Model

Human endothelial cells (hCMEC/D3) were cultured in 96 flat-bottom, black-well microplates (Corning, New York, NY, USA) pre-coated with 100 μg/mL rat tail collagen. Cells were seeded at 0.5 × 10^4^ cells/mL in a final volume of 250 μL of complete EBM2 medium per well. Fully confluent cells were irradiated at 25 Gy or sham-irradiated. After 2 days, a mixture of 200 µL of recalcified whole human blood containing FITC-fibrinogen (130 µg/mL) with VTA (0, 2.5, 5 or 10 µg/mL in normal saline) was added by a multichannel pipettor to each well. To evaluate the reproducibility of the assay, at least a triplicate of each group was tested with the same blood donor for irradiated and sham-irradiated cells. After 10 min of incubation, plates were inverted and tapped to remove excess blood. Cells were thoroughly washed three times with 500 µL PBS 1× and fixed with ethanol (50% *v*/*v*) in PBS. The level of deposited fibrin in each well was first assessed via fluorescence microscopy (EVOS Cell Imaging Systems, Thermo Fisher, Massachusetts, USA) and the corresponding integrated density per field of view determined using NIH Image J [33]. In addition, the FITC signal of deposited fibrinogen was read by PHERAstar microplate reader (BMG Labtech; Offenburg, Germany). The protocol was set up with excitation wavelength at 485 nm and emission at 520 nm. For each experiment, the focal height and gain adjustment were performed before reading. Single and well-scanning measurements including matrix, orbital and spiral were tested to establish the best scan mode. A single center bottom reading was consequently selected as a detection mode. To prevent and minimize auto-fluorescent signals (e.g., from the empty microplate, over-confluent cells and irradiated cells) and to reduce intra-experimental bias, values were first corrected to the blank (empty wells) and then divided by the average of the negative control wells (no coaguligand) for each plate separately to give final integrated FITC intensity values.

### 4.10. Statistical Analysis

Data analyses were performed using Prism 8.4.3 (GraphPad Software Inc., CA, USA). Values represent mean ± SEM of at least three independent experiments unless otherwise stated. Two-way ANOVA was employed to calculate statistical differences with Tukey’s post-hoc analysis. *p*-value was set to less than 0.05.

## Data Availability

Not applicable.
